# Case series of selenium insufficiency among infants with intestinal failure

**DOI:** 10.1016/j.intf.2026.100352

**Published:** 2026-01-12

**Authors:** Darren Bodkin, Alisha Hemani, Jessica Miller, Jennifer Fraysur, Erica Fuchs

**Affiliations:** a Section of Neonatal Perinatal Medicine, Department of Pediatrics, University of Oklahoma Health College of Medicine, USA; b University of Oklahoma Health, College of Medicine, USA; c Department of Pharmacy, Oklahoma Children’s Hospital OU Health, USA

**Keywords:** Selenium, Intestinal failure, Parenteral nutrition

## Abstract

**Background:**

Selenium (Se) is an essential trace element incorporated into ‘selenoenzymes’ that are necessary for immune, thyroid and antioxidant responses. Infants with intestinal failure (IF) require prolonged administration of parenteral nutrition (PN). This report presents a series of infants with IF who demonstrate biochemical Se deficiency despite PN supplementation, which normalized following dose escalation.

**Case Series:**

Patient 1 was born at 25 weeks’ gestation, birthweight 870 g, with intestinal malrotation and colonic atresia. Patient 2 was born at 28 weeks’ gestation, birthweight 1130 g, with multiple congenital anomalies. Patient 3 was born at 34 weeks’ gestation, birthweight 3030 g with gastroschisis and midgut volvulus requiring significant bowel resection. Their serum Se levels ranged from 56 to 93 (reference range 100–340 µg/L) prior to dose changes.

**Conclusion:**

Preterm birth, bowel resection and intestinal failure are risk factors for Se deficiency. High quality studies of PN Se supplementation in preterm infants with IF are needed to determine the optimal dose for this high-risk population.

## Introduction

Pediatric intestinal failure (IF) is the reduction in functional gastrointestinal mass, resulting in dependence on supplemental parenteral nutrition [1]. Short bowel syndrome (SBS) is a subcategory of IF, characterized by insufficient absorptive surface area due to loss of the small bowel that results from either congenital absence or surgical resection [1]. SBS has an estimated incidence 22.1 per 1000 NICU admissions, accounting for 0.7 % among infants with birthweight less than 1500 g [1]. Necrotizing enterocolitis (NEC) is the most common cause of SBS in neonates; congenital causes of SBS include intestinal atresia, gastroschisis, volvulus, and Hirschsprung’s disease. While parenteral nutrition (PN) has improved outcomes of neonates with SBS, PN dependency has adverse associations such as PN-associated liver disease, macronutrient and micronutrient deficiencies, with a higher risk of growth failure. Additionally, children on home PN may have an increased risk of psychopathology due to a lower quality of life [2]. The aim of this report is to highlight the occurrence of biochemical Se deficiency among three infants with IF on PN and the concern of suboptimal formulation to meet their metabolic demands.

Selenium (Se) is an essential trace element necessary for production of selenoproteins, which are necessary for optimal cellular antioxidant capacity [3], [4]. Fetal liver Se storage peaks in the third trimester of pregnancy, so preterm infants are inherently deficient relative to term infants. Furthermore, there is a paucity of data for Se needs for periviable neonates 22–25 + 6 weeks [5], [6]. This population is at high risk of complications of prematurity such as severe NEC requiring bowel resection. These surgical procedures are associated with oxidative stress and pro-inflammatory mediators. This creates an imbalance of redox homeostasis and potentially curtails normal cell growth and intestinal adaptation [7]. Intestinal adaptation includes structural and functional changes such as epithelial cell proliferation and transporter protein function needed for adequate nutrient absorption [8]. A specific selenoprotein, Glutathione peroxidase 2 (GPX2) is essential for mucosal integrity. Therefore, Se optimization in PN may be an underutilized trace element in post-surgical intestinal recovery [3]. Although research supporting a higher dose of Se in infant PN has led to a change of practice in Europe, there has been a significant delay in adopting these recommendations in the United States.

Currently the American Society of Parenteral and Enteral Nutrition (ASPEN) recommended dose of Selenium in TPN is 2 µg/kg/day for preterm and term infants. In 2012 ASPEN recommended 1.5–4.5 µg/kg/day for preterm infants. However, in our cohort, we have seen, Se deficiency in infants with IF that require PN despite receiving the recommended supplemental dosage. Nationally many centers give on average approximately 2 µg/kg/day. This contrasts with the European Society for Pediatric Gastroenterology Hepatology and Nutrition (ESPGHAN) and the European Society for Clinical Nutrition and Metabolism (ESPEN) recommendation of 7 µg/kg/day of Se for preterm infants and 2–3 µg/kg/day for infants and children [12]. These recommendations were adopted from studies showing higher doses needed to sustain target serum Se levels, while avoiding adverse effects [9]. Reference ranges for serum selenium varies with hospital laboratory data due to regional differences in population data, and testing methods. Although this is beyond the scope of this report, we see an opportunity for creating a gestational age specific national reference standard using a multi-disciplinary collaborative approach in clinical research. While the exact phenotype of selenium deficiency is not yet agreed upon, recent animal data suggests that deficiency would be seen among infants with growth failure, bronchopulmonary dysplasia with biochemical low Se levels [10]. To date, there has not been a definitive study that has evaluated the Se dosing needs of preterm infants with intestinal failure. We present a case series of infants with IF who have Se deficiency despite PN with recommended dose.

## Case Series

### Patient 1

Patient 1 is an extremely low birthweight (ELBW) male born at 25 weeks, with a birthweight of 870 g and a hospital course complicated by spontaneous intestinal perforation with a peritoneal drain placed at day of life (DOL5). On DOL 25 the neonate was transferred to our level IV neonatal intensive care unit for neurosurgical evaluation due to concern for hydrocephalus. After consultation with pediatric gastroenterologist, a HIDA scan was performed, and a diagnosis of biliary atresia was excluded. This neonate developed PN associated cholestasis, peak direct bilirubin of 20.6 mg/dl. The patient was subsequently transitioned to Omegaven lipids and alternate day trace element supplementation. Trophic feeds of maternal milk was initiated per nutrition protocol but was not tolerated. Upper GI with small bowel follow through was negative for obstruction or malrotation. Several attempts to administer enteral feeds were not tolerated, as the patient developed abdominal distension. Operative findings at exploratory laparotomy included: 1) colonic atresia at the splenic flexure necessitating the creation of a Santulli colostomy; 2) intestinal malrotation requiring Ladd’s procedure; and 3) gastrostomy tube placement.

**Fig. 1:**
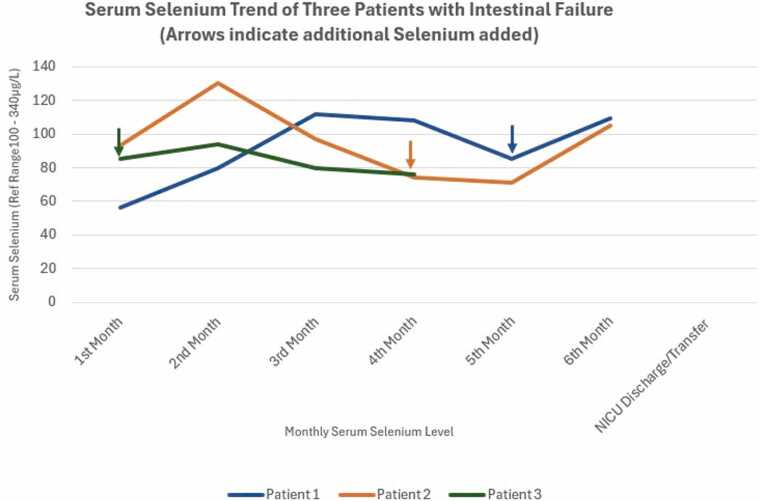
Serum selenium level trend of three infants with intestinal failure during hospital neonatal intensive care unit admission. Selenium reference range is 100–340 (µg/L).Downward point arrows, color coded for each patient, indicate when additional selenium was added to the parenteral nutrition.

Trophic feeds were restarted 1 month post op (∼3 months post menstrual age) at 10 ml/kg/day of maternal milk or donor breastmilk with slow advancement per intestinal rehabilitation guidelines. Se level was low despite PN containing the recommend 2 µg/kg/day. The initial Se level was 56 µg/L (Reference range 100–340 µg/L, Table 1) while receiving the standard PN dose 2 µg/kg/day daily. The monthly serum Se level improved despite limiting trace element supplementation for the first three months of admission. After resolution of cholestasis, we resumed daily trace element supplementation at the third month of admission, with additional Se given for an approximate total daily dose of 4 µg/kg/day Se on the fifth month (see downward arrow on Fig. 1). In three months, the serum Se level was within the normal range with the highest level at 112 µg/L. The infant was discharged home on 20 calorie Alfamino feeds via GT at 120 ml/kg/day.

**Table 1:** Patients 1–3 demographics, initial selenium level and PN adjustments.

**Patient**	**Gender**	**Gestational Age at Birth**	**Birthweight (grams)**	**Etiology of Intestinal Failure**	**Non GI Anomalies**	**Duration of TPN only before Feed Initiation (Days)**	**Serum Selenium at first check (µg/L) Range 100–340**	**Selenium Change in TPN**	**Outcome**
1	Male	25wks	870	Malrotation with colonic atresia. Surgical care: Ladd’s procedure, Santulli colostomy & GT. Complications: abdominal compartment syndrome, incarcerated incisional hernia	None	136	56	Additional Se for total ∼ 4 µg/kg Se	Discharged home GT feed F/u Intestinal Rehab
2	Female	28wks + 4 days	1130	Imperforate anus. Surgical care: creation of end colostomy and mucous fistula and GT.	Type C Tracheoesophageal fistula, cloacal anomaly Ambiguous Genitalia	60	93	Additional Se for total ∼4 µg/kg/day	Discharged home GJ feed F/u Intestinal Rehab
3	Male	34wks+ 6 days	3030	Gastroschisis, and volvulus. Surgical care: Jejunostomy, mucous fistula & GT	none	114	85	Additional Se for total ∼4 µg/kg	Transferred to Rehabilitation Facility GT feed and TPN

### Patient 2

Patient 2 is an ELBW female at 28weeks & 4 days 1130 g with multiple congenital anomalies including VACTERL association – tracheoesophageal fistula, imperforate anus and cloacal anomaly. Initial surgical operation included loop colostomy and gastrostomy tube (GT), followed by another laparotomy resulting in an end colostomy and mucous fistula. The infant was then transferred to our NICU for multi-disciplinary surgical care and prolonged PN dependence in the setting of a suspected colovesical fistula. The infant was PN dependent and developed PNALD with peak direct bilirubin 13.6 mg/dl. Omegaven lipids were administered and trace elements given on alternate days. On days the infant did not get trace elements, they received Se at 2 µg/kg/day. Cholestasis resolved and patient resumed soybean, medium chain triglyceride, olive and fish oil (SMOF) lipids in PN nutrition. Enteral feeds via GT were started at 3 months PMA although there were multiple interruptions due to recurrent surgical interventions during the admission.

Initial Se level was 90 µg/L (Table 1) with PN containing the recommended 2 µg/kg/day of Se. Monthly Se levels showed a steady decrease in serum levels with the lowest being 71 µg/L. To optimize Se content, beginning at the fourth month of admission every 48 h TPN contained an additional 2 µg/kg/day for a total of 4 µg/kg/day (see downward arrow on Fig. 1). During 3 months of monitoring the serum Se peaked at 105 µg/L. During the NICU hospitalization the GT was converted to a GJ, and the infant was discharged home receiving 110 ml/kg/day of term formula at 20 kcal.

### Patient 3

Patient 3 is a late preterm male, 34weeks & 6 days, 3030 g with gastroschisis, and volvulus who developed short bowel syndrome after significant bowel resection and the creation of jejunostomy and mucous fistula of transverse colon. The patient’s ileocecal valve was resected with approximately 40 cm of small bowel remaining. Mucous fistula feeding with formula attempted, however due to feeding intolerance, PN dependence and need for intestinal rehabilitation care he was transferred to our NICU. During this admission another laparotomy was indicated which found volvulus of the small bowel requiring further resection of 5 cm small bowel, colon within normal appearance, post op abdomen left open and bowel in discontinuity and silo bag. The abdomen was examined again within 48hrs, and the small bowel was found to be viable. At the conclusion of the operation, the patient had a gastrostomy tube, 35 cm small bowel from duodenum to end jejunostomy and mucous fistula from the transverse colon. At 12 weeks old, (∼2 weeks post op), trophic feeds via the GT were initiated. Contrast study of mucous fistula was reassuring. At 3-week post op we commenced mucous fistula refeeding using Elecare at 5 ml/kg/day as continuous infusion. Progression of feeding was slow given the high ostomy output. This patient developed PNALD, with a peak direct bilirubin of 2.3 mg/dL. Parenteral nutrition adjustments included switching from SMOF to Omegaven lipids. The direct hyperbilirubinemia resolved quickly, and the patient resumed SMOF lipids. Trace element supplementation continued daily due to low baseline numbers.

Overall, the patient received PN for 4 months with additional Se. The initial Se level was 85 µg/L (Table 1), below lower end goal of 100 µg/L. This was addressed on the first month of admission with increasing the PN Se dose to 4 µg/kg/day (see downward arrow notation on Fig. 1). Subsequent monthly checks of Se level remained below target. The Se level peaked at 94 µg/L prior to transfer to the pediatric floor. The patient was ultimately discharged to a chronic rehabilitation facility with predominantly PN and trophic volume enteral feed of breastmilk via GT.

## Discussion

This case series illustrates that infants with bowel resection, and prolonged PN dependence are at high risk of Se deficiency despite receiving the recommended 2 µg/kg/day. In all three cases, the infants were delivered preterm, with significant bowel resection or prolonged delay in initiating enteral feeding, which are known risk factors for micronutrient deficiencies. Selenium deficiency is difficult to identify in neonates, and in our cohort we did not formally screen infants for this. In our series the 3 infants were prescribed additional Se above the standard dose of 2 µg/kg/day, for a total daily intake of ∼ 4 µg/kg/day. This is still well below the recommendation of 7 µg/kg/day by ESPGHAN and ESPEN. Unsurprisingly, serum levels in our cohort never approached the upper limits of normal.

IF is a rare but devastating condition where gastrointestinal absorptive function is insufficient to sustain life and therefore requires complex medical management by a multidisciplinary team [2]. This team is more commonly referred to as the intestinal rehabilitation program (IRP). They are critical for promotion of intestinal adaptation and enteral autonomy, decreased need for intestinal transplantation, and prevention of micronutrient deficiencies [11]. Overall, they have made great improvements in the quality of life for children. However, discussion on Se has not gained much attention nationally.

Se is an essential micronutrient which contributes to the endogenous systems of immunity and redox regulation to mitigate oxidative stress [5], [6]. Morbidity among preterm infants is increased from diseases of oxidative injury. Therefore, adequate Se in nutrition is hypothesized to mitigate the diseases of preterm oxidative injury, such as chronic lung disease and retinopathy of prematurity. Although there is data to support higher Se dose in PN, the North American, supplementation guidelines, ASPEN are conservative relative to European ESPEN guidelines [12]. A retrospective study of neonates on greater than 2 weeks of PN nutrition showed 54 % with selenium deficiency with this deficit worse in preterm infants [13]. The largest randomized controlled trial with infants birthweight < 1500 g by Darlow et al. showed that as much as 7 µg/kg/day was needed for attaining mean serum levels of healthy term infants. This higher dose for preterm infants was not associated with toxicity. This led to a clinical practice change in Europe, with ESPEN 2018 guidelines making this a strong recommendation with strong consensus [12]. In North America, ASPEN guidelines have not increased recommendations for clinicians and in many institutions Se dose is limited to 2 µg/kg/day.

In all three cases in this report, the infants received additional Se at a total of ∼4 µg/kg/day. Serial serum monitoring revealed an incremental increase in patient 1 to the lower limit of normal. The serum Se for patient 3 never increased to a normal range. There is a paucity of data evaluating the specific parenteral needs of infants with intestinal failure. Se insufficiency may be an unrecognized modifiable factor that has the potential to improve infant morbidity in preterm infants with intestinal injury and significant bowel resection.

## Conclusion

Infants with long term PN dependence are at high risk of biochemical Se deficiency. The recommended Se content in PN of 2 µg/kg/day is likely to be inadequate for preterm infants with intestinal failure, oxidative stress and bowel resection. Routine biochemical testing among infants at high risk of Se deficiency with augmentation of daily intake might limit morbidity due to reactive oxidative species. Future clinical studies are needed to evaluate the optimal dose of Se in PN for infants with intestinal failure.

## CRediT authorship contribution statement

**Erica Fuchs:** Writing – review & editing, Formal analysis, Data curation. **Jennifer Fraysur:** Writing – review & editing, Writing – original draft, Formal analysis, Data curation. **Jessica Miller:** Writing – review & editing, Writing – original draft, Formal analysis, Data curation. **Alisha Hemani:** Writing – review & editing, Writing – original draft, Data curation. **Darren Bodkin:** Writing – review & editing, Writing – original draft, Supervision, Data curation, Conceptualization.

## Patient's/ Guardian's consent

Informed consent was obtained from the patients, parents/legal authorized representative.

## Ethical approval

This study received institutional review board approval #17980 dated February 7th 2025.

## Funding

This research did not receive any specific grant from funding agencies in the public, commercial or not-for-profit sectors.

## Declaration of Competing Interest

The authors declare that they have no known competing financial interests or personal relationships that could have appeared to influence the work reported in this paper.
